# Successful Cochlear Implantation for Intracochlear Fibrosis

**DOI:** 10.7759/cureus.43042

**Published:** 2023-08-06

**Authors:** Mustafa A Khawaja, Mahomud AlRamahi, Mohammad Hashlamoun, Adel K Adwan

**Affiliations:** 1 General Practice, School of Medicine, Al-Quds University, Jerusalem, PSE; 2 Radiology, Augusta Victoria Hospital, Jerusalem, PSE; 3 Clinical Audiology, The Emirates Hearing Center, Hebron, PSE; 4 Otorhinolaryngology, School of Medicine, Al-Quds University, Jerusalem, PSE

**Keywords:** scala tympani, otology, cochlear implantation, inracochlear fibrosis, sensorineural hearing loss

## Abstract

Intracochlear fibrosis is a rare disorder that can lead to hearing loss and make cochlear implantation challenging. The etiology of intracochlear fibrosis is diverse, including infections, inflammation, and past surgical procedures. The condition causes ossification and scar tissue growth within the cochlea, leading to progressive obstruction of the cochlear turn. High-resolution computed tomography (HRCT) and magnetic resonance imaging (MRI) are sensitive diagnostic modalities for fibrosis and ossification. There is a paucity of information in the literature regarding cochlear implantation during the fibrotic stage.

This case report discussed the presentation, diagnosis, and surgical management of intracochlear fibrosis in a patient with a history of sudden and severe hearing loss. A 44-year-old female patient with a 20-year history of sudden profound sensorineural hearing loss (SNHL) in both ears was successfully treated with cochlear implantation. Thorough preoperative planning for cochlear implantation, including HRCT and MRI cochlear protocol, is crucial for identifying intracochlear fibrosis, which can be missed on routine audiometry. She underwent a surgery for right cochlear implantation using postauricular approach. Drilling was done to the round window niche, and we removed an abnormal, chalky white bone we encountered by continuing to drill this abnormal bone following the scale tympani until we identified the opening of the scala tympani, then we inserted the cochlear implant device. She was doing well on the subsequent post-operative follow-up.

Intracochlear fibrosis treatment with cochlear implantation has proven successful in several studies. Audiologic outcomes vary with time to implantation, so an early attempt should be made for cochlear implantation. Follow-up is important to monitor auditory outcomes

## Introduction

Intracochlear fibrosis is a rare disorder that can be linked to various events, including infections, inflammation, or past surgical procedures. This condition causes ossification and scar tissue growth within the cochlea, which can lead to hearing loss and make cochlear implantation complicated. Labyrinthitis ossificans may develop after meningitis, labyrinthitis, temporal bone damage, stapedectomy, labyrinthectomy, autoimmune sensorineural hearing loss (SNHL), and lateral skull base surgery, particularly the translabyrinthine approach [1,2]. Even weeks after an insult, obliteration can happen, but it's not clear which patients it will affect [2]. Cochlear ossification is a progressive process that progresses from acute inflammation to fibrosis, then to neo-ossification, and finally to cochlear turn obstruction [3]. It has been reported that the combination of high-resolution computed tomography (HRCT) and magnetic resonance imaging (MRI) imaging techniques in the assessment of early ossification is sensitive in the 90% range. T2-weighted MRI (T2MRI) is a diagnostic modality that visualizes fluid distributions within the cochlea and can therefore detect narrowing or obliteration of the cochlear lumen caused by fibrosis or ossification [4,5]. Nonetheless, there is a scarcity of information regarding cochlear implantation during the fibrotic stage. We identified a study that compared the efficacy of microdissection, laser vaporization, and various dilation procedures for the treatment of intracochlear fibrosis [1]. We hope that by sharing this case, we can add to the growing body of evidence about how to treat intracochlear fibrosis and show how important it is for people with complex hearing problems to have individualized treatment plans. We present the case of a 44-year-old female patient with a 20-year history of sudden profound hearing loss in both ears. She was treated successfully with cochlear implantation.

## Case presentation

**Figure 1:**
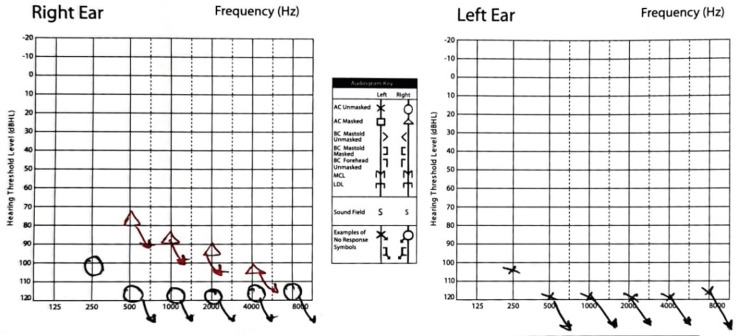
Audiogram test for the mentioned patient revealing profound-degree SNHL on both sides. SNHL: sensorineural hearing loss

A 44-year-old woman presented to the otology clinic with a history of 24 years of sudden hearing loss that started in her left ear and, after one year, moved to her right ear with a profound degree of SNHL with an unclear etiology. She wore hearing aids only for one week before taking them off, as she did not get any benefit out of them. She was asthmatic with no other medical issues. She had her hearing test done, which showed bilateral profound SNHL (almost dead ears) (Figure 1), and she has been advised to go for cochlear implantation, especially since she has excellent expressive language skills. She had agreed to the cochlear implant.

**Figure 2:**
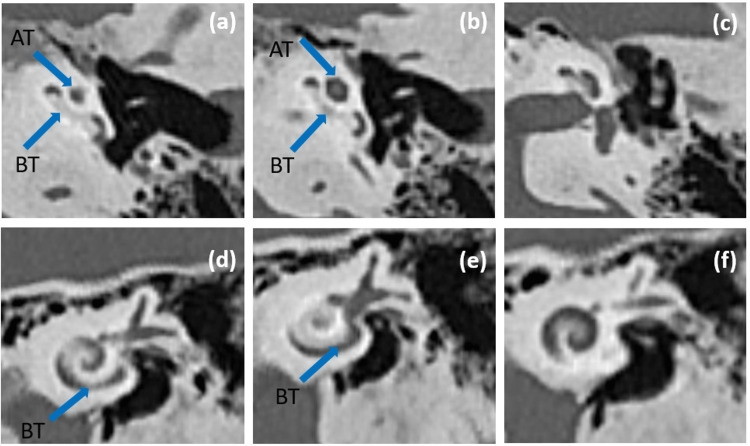
(a-c): axial CT scan of the left cochlea, and (d-f): coronal CT scan of the left cochlea. Both views revealed the cochlea with relatively confluent hyperdense plaque of mineralization in the basal and middle turns just proximal to the round window. AT: apical turn. BT: basal turn.

Clinical data for preoperative planning for cochlear implantation include a high-resolution CT scan for both temporal bones with coronal reconstruction acquired without IV contrast, as well as an MRI cochlear protocol. On bilateral CT scan, the cochlea was found to have relatively confluent hyperdense plaque of mineralization in the basal and middle turns just proximal to the round window, which is consistent with the Symons and Fanning grading system for retrofenestral otosclerosis grade 2c (Figure 2). On MRI of both sides, there is relatively narrowing in the cochlear turns, however, with preserved signal intensity. The facial nerve, along with the facial nerve canal, has a normal appearance and course. Otic capsule visibly normally mineralized. Vestibular aqueduct noted with normal size. No middle ear cavity opacifications. The scutum and tegmentum tympani are preserved. Ossicles were in normal alignment. Mastoid air cells were well-formed.

**Figure 3:**
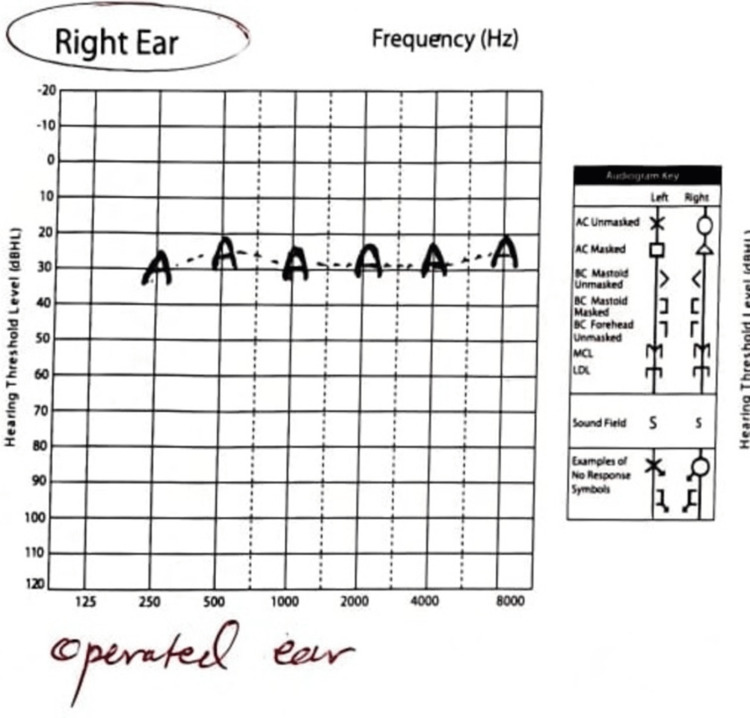
Post-operative audiological assessment for the right ear with spectacular result.

Data collected from the clinical support team in Austria showed that the scala tympani were ossified for an insertion depth of a bit more than 180 degrees of angular depth, were the advice was to drill a cochleostomy in the scala vestibule instead. One month later, she had her right cochlear implantation successfully done on her right side. Lazy retroauricular incision done, with mastoidectomy posterior tympanotomy approach. 1mm inferior to the pyramidal process, drilling was done to the round window niche, and abnormal, chalky white bone was encountered. We continued drilling this abnormal bone following the scale tympani about 5-8mm then we identified the opening of the scala tympani. After that a MED-EL device (Sonata 2 F28; MED-EL GmbH, Innsbruck, Austria) with full insertion was implanted. One, three, and six months postoperatively, she had her audio processor switched on with a marvelous auditory outcome within two consecutive sessions, and she is doing excellent (Figure 3). 

## Discussion

This case report highlights the importance of thorough preoperative planning for cochlear implantation, which includes an HRCT scan for both temporal bones with coronal reconstruction acquired without IV contrast, as well as an MRI cochlear protocol. These imaging modalities can help identify intracochlear fibrosis, which can be missed on routine audiometry.

There are numerous causes of intracochlear fibrosis; bacterial meningitis is the most prevalent cause of labyrinthitis ossificans and profound acquired bilateral SNHL in childhood, with the highest incidence of hearing loss occurring in meningitis due to Streptococcus pneumoniae (31%) [6]. Another common cause for itracochlear fibrosis is lateral skull base surgery, especially the translabyrinthine approach (eg; vestibular schwannoma resection) [1,7], where we found a paucity of literature that reported the simultaneous vestibular schwannoma tumor resection and cochlear implantation as a viable and even preferential option in certain clinical situations [7,8]. A rare but likely underrecognized cause of hearing loss that may cause itracochlear fibrosis/ossifications is autoimmune inner ear disease. It has been reported that about 54% of patients who undergo cochlear implantation due to autoimmune inner ear disease were found to have intracochlear fibrosis/ossification intraoperatively [9,10]. In our case, there was no clear etiology identified for the patient’s sudden SNHL, which is likely to be referred to as idiopathic intracochlear fibrosis [1].

One interesting thing in our case comes from the fact that ossification previously was thought to contraindicate cochlear implantation because of possible mechanical obstruction and uncertainty about the level of function that could be achieved by stimulating an ossified cochlea [11]. Histopathologic studies have demonstrated that the most common region of cochlear ossification, regardless of the etiology, is the basal turn of the scala tympani [1,11]. In our case, basal and middle turns just proximal to the round window were involved. Cochlear implantation in the presence of intracochlear fibrosis is a challenging technique requiring meticulous planning and execution. Various surgical procedures, including drilling a cochleostomy in the scala vestibuli, establishing a subperiosteal pocket, and utilizing a laser to build a route through the fibrotic tissue, have been documented in the literature [1]. In our case, we have been advised to drill a cochleostomy in the scala vestibule instead of scala tympani due to ossification, but we followed the preferred approach, which was called “postauricular approach”, with complete mastoidectomy and posterior tympanotomy, then round window insertion, and cochleostomy into the scala tympani performed when round window approach was not possible [1], but in our case, 1mm inferior to pyramidal process drilling to the round window niche was done, and abnormal, chalky white bone was encountered. We continued drilling this abnormal bone following the scale tympani about 5-8mm then we identified the opening of the scala tympani. A full insertion of the cochlear implant device was done. It's a well-known fact that if the cochlear nerve is preserved and fibrosis or ossification of the cochlea does not cause a severe barrier to electrode insertion, effective stimulation is achievable [4].

Whether fibrosis removal or dilation was performed, there is no difference in the pure tone average (PTA) (p = 0.76), according to a previous study. Those who waited the longest to get surgery had the worst outcomes: four months after meningitis and 24 months after idiopathic abrupt SNHL [1]. In our case, we performed the removal of fibrosis, where we continued drilling the abnormal, chalky white bone following the scale tympani. On subsequent follow-up, she was well and doing excellent with a striking auditory outcome.

## Conclusions

Intracochlear fibrosis is a rare condition, in which the cochlea is found to be ossified and/or fibrotic. It can occur post-meningitis, or due to a systemic autoimmune disease causing inner ear disease. It can also result from a translabyrinthine approach in temporal bone surgery. If there is no clear etiology, it is called idiopathic intracochlear fibrosis causing sudden SNHL. It can be diagnosed using a temporal bone CT scan and MRI, which can reveal fibrosis and mineralization within the cochlear turns, and narrowing, respectively. A successful treatment option is cochlear implantation after the limited fibrosis is removed or dilated if it is too extensive to permit implantation. According to several studies, good cochlear implantation audiologic outcomes vary with time to implantation, so intervention should be made as early as possible. The main goal after cochlear implantation is strict follow-up with the patient. We need further studies on the intracochlear fibrosis subject in the future, in order to increase awareness among physicians and to provide early diagnosis and treatment as soon as possible.

## References

[1] Maxwell AK, Kahane JB, Mehta R, Arriaga MA (2023). Cochlear implantation through intracochlear fibrosis: a comparison of surgical techniques. Cochlear Implants Int.

[2] Erbele ID, Miller LS, Mankekar G, Morel CE, Anderson DT, Son LS, Arriaga MA (2020). Cochlear enhancement may precede cochlear obliteration after vestibular schwannoma excision. Otol Neurotol.

[3] Carswell V, Crowther JA, Locke R, Taylor W, Kontorinis G (2019). Cochlear patency following translabyrinthine vestibular schwannoma resection: implications for hearing rehabilitation. J Laryngol Otol.

[4] Hill FC, Grenness A, Withers S, Iseli C, Briggs R (2018). Cochlear patency after translabyrinthine vestibular schwannoma surgery. Otol Neurotol.

[5] Philippon D, Bergeron F, Ferron P, Bussières R (2010). Cochlear implantation in postmeningitic deafness. Otol Neurotol.

[6] Tinling SP, Colton J, Brodie HA (2004). Location and timing of initial osteoid deposition in postmeningitic labyrinthitis ossificans determined by multiple fluorescent labels. Laryngoscope.

[7] DeHart AN, Broaddus WC, Coelho DH (2017). Translabyrinthine vestibular schwannoma resection with simultaneous cochlear implantation. Cochlear Implants Int.

[8] Sanna M, Piccirillo E, Kihlgren C, Cagliero G, Guidi M, Saleh E (2021). Simultaneous cochlear implantation after translabyrinthine vestibular schwannoma resection: a report of 41 cases. Otol Neurotol.

[9] Goh X, Muzaffar J, Bance M (2022). Cochlear implantation in systemic autoimmune disease. Curr Opin Otolaryngol Head Neck Surg.

[10] Lee J, Biggs K, Muzaffar J, Bance M, Monksfield P (2021). Hearing loss in inner ear and systemic autoimmune disease: a systematic review of post-cochlear implantation outcomes. Laryngoscope Investig Otolaryngol.

[11] Marchioni D, Soloperto D, Bianconi L, Guarnaccia MC, Genovese E, Presutti L (2016). Endoscopic approach for cochlear implantation in advanced otosclerosis: a case report. Auris Nasus Larynx.

